# Cellular and molecular mechanisms underlying obesity in degenerative spine and joint diseases

**DOI:** 10.1038/s41413-024-00388-8

**Published:** 2024-12-11

**Authors:** Qian Xiang, Zhenquan Wu, Yongzhao Zhao, Shuo Tian, Jialiang Lin, Longjie Wang, Shuai Jiang, Zhuoran Sun, Weishi Li

**Affiliations:** 1 Department of Orthopaedics, Peking University Third Hospital, Beijing, China; 2 Engineering Research Center of Bone and Joint Precision Medicine, Ministry of Education, Beijing, China; 3 Beijing Key Laboratory of Spinal Disease Research, Beijing, China

**Keywords:** Pathogenesis, Diseases

## Abstract

Degenerative spine and joint diseases, including intervertebral disc degeneration (IDD), ossification of the spinal ligaments (OSL), and osteoarthritis (OA), are common musculoskeletal diseases that cause pain or disability to the patients. However, the pathogenesis of these musculoskeletal disorders is complex and has not been elucidated clearly to date. As a matter of fact, the spine and joints are not independent of other organs and tissues. Recently, accumulating evidence demonstrates the association between obesity and degenerative musculoskeletal diseases. Obesity is a common metabolic disease characterized by excessive adipose tissue or abnormal adipose distribution in the body. Excessive mechanical stress is regarded as a critical risk factor for obesity-related pathology. Additionally, obesity-related factors, mainly including lipid metabolism disorder, dysregulated pro-inflammatory adipokines and cytokines, are reported as plausible links between obesity and various human diseases. Importantly, these obesity-related factors are deeply involved in the regulation of cell phenotypes and cell fates, extracellular matrix (ECM) metabolism, and inflammation in the pathophysiological processes of degenerative spine and joint diseases. In this study, we systematically discuss the potential cellular and molecular mechanisms underlying obesity in these degenerative musculoskeletal diseases, and hope to provide novel insights for developing targeted therapeutic strategies.

## Introduction

Intervertebral disc degeneration (IDD), ossification of the spinal ligaments (OSL), and osteoarthritis (OA) are most common degenerative spine and joint diseases in the older adults.^1–4^ IDD is regarded as a major cause of discogenic lower back pain that can cause physical activity limitation or even disability globally.^5–7^ OSL is a major contributor to degenerative spinal stenosis that can impact the function and life quality severely and substantially in the aged population.^2,8^ Osteoarthritis is a common joint disorder leading to chronic pain and physical disability that affects approximately 3.3%–3.6% of the worldwide population.^9^ However, these degenerative musculoskeletal disorders are multi-factorial and complex, involving genetic and environmental factors. Tremendous efforts have been put into the investigation of theses disease pathogenesis and development. Unfortunately, the cellular and molecular mechanisms of the degenerative spine and joint diseases remain elusive. Further research is needed to uncover the specific mechanisms of these diseases to develop novel therapeutic strategies. Although aging is the most well-known risk factor for many degenerative musculoskeletal diseases, more and more studies have reported the essential roles of obesity in the pathogenesis and progression of these diseases.^4,9–12^

Obesity is a common and complex metabolic disorder characterized by excessive accumulation of adipose tissue or abnormal adipose distribution in the body. The prevalence of obesity is increasing globally, accompanied by a parallel rise in musculoskeletal disorders.^13^ In recent years, there is a profound interest to uncover the underlying links between obesity and obesity-related degenerative musculoskeletal diseases. Studies have reported that obesity-induced mechanical stress is a critical risk factor for obesity-related pathology.^14,15^ Interestingly, the human adipose tissues can also function as an active endocrine organ that releases many biologically active molecules termed as “adipokines”, which affect metabolic and inflammatory processes to play critical roles in obesity-related diseases.^16^ Besides, the occurrence of obesity is also characterized by lipid metabolism disorder that is pathologically linked to obesity-related diseases.^17^ Obesity-mediated lipid metabolism disorder, dysregulated pro-inflammatory adipokines and cytokines, are implicated in multiple human diseases pathogenesis, including degenerative spine and joint diseases.^9,18^

A deeper understanding of the association between obesity and degenerative diseases might help to decipher the complex pathogenesis of diseases to develop novel therapeutic strategies. In this work, we systematically discuss the critical functions and mechanisms of mechanical stress, different adipokines, obesity related lipid metabolism disorder, and pro-inflammatory cytokines in degenerative spine and joint diseases. We aim to comprehensively review the underlying mechanisms of obesity in these degenerative musculoskeletal diseases, and hope to provide novel insights for developing clinical treatments.

## Obesity and IDD

Intervertebral disc (IVD) is a critical fibrocartilaginous structure necessary for absorbing mechanical load and also provides flexibility for the spine. A normal IVD consists of nucleus pulposus (NP), annulus fibrosus (AF), as well as cartilaginous endplates (EP). Pathologically, the NP is replaced by fibroblast-like phenotype cells with an imbalance of catabolism and anabolism of disc extracellular matrix (ECM) in the degeneration progression.^19^ The IDD process is also characterized by annulus fibrosus disorder or rupture, and cartilaginous endplates sclerosis or calcification.^20^ Accumulating evidence reveal that IDD is closely associated with the disc cell phenotypes and cell fates, ECM metabolism, and inflammation response.^21^ However, the specific mechanisms of IDD pathogenesis and development remain unclear.

In recent years, more and more studies have focused on the roles and functions of obesity in degenerative disc diseases.^22–24^ Obesity-induced mechanical stress is regarded as a critical risk factor for IDD pathogenesis.^14,25,26^ Besides, obesity is characterized by enlarged adipose tissue or abnormal adipose distribution in the body. Moreover, the adipose tissues have endocrine functions that secret a group of biologically active molecules called adipokines. Additionally, obesity-related lipid metabolism disorder and dysregulated inflammatory cytokines have been investigated in various obesity-related diseases.^9,16,18^ We review the association between IDD and obesity-related factors in the following text to elucidate the potential mechanisms of IDD.

### Mechanical stress meditates IDD

The intervertebral disc is subjected to complex mechanical stress in daily life and previous research has demonstrated that abnormal mechanical stress might increase the risk of IDD occurrence.^27^ A previous study has reported that the lumbar disc was subjected to a pressure of 0.5 MPa in relaxed standing, a pressure of 2.3 MPa with a 20 kg-weight in a flexed position.^28^ Obesity and overweight cause an increased level of mechanical loading on the spine disc, and thus contribute to IDD potentially.^14^ Coppock et al. found a close relationship between body mass index (BMI) and in vivo IVD deformation, suggesting that obesity may change the mechanical response of the disc.^26^ More recently, Singh et al. reported that an increased BMI was closely associated with excessive stress and deformation of lower spine, which might predispose individuals to IDD.^14^ Particularly, NP was more susceptible compared with AF in these conditions. A great number of studies have reported that heightened mechanical stress increased the apoptosis in NP cells, ECM metabolism disorder, and contributed to the remodeling of the disc during degeneration process.^29,30^ Additionally, abnormal mechanical stress was found to induce the endplate cartilage degeneration to accelerate IDD progression.^31,32^ Excessive mechanical stress also exerted damaging effects on the annulus fibrosus to further promote IDD development.^33^ However, the studies focused on specific function and mechanism of obesity-meditated mechanical stress in IDD are limited, and further related investigation is needed.

### Adipokines function in IDD

Obesity also contributes to IDD by biological mechanisms apart from mechanical means. Most importantly, the biologically active adipokines secreted by adipose tissues have played key roles in the pathophysiology of IDD. Leptin is a key adipokine mainly secreted by white adipose tissues and exerted diverse biological functions.^34^ Various research has focused on the function and mechanism of leptin in obesity-related IDD. A previous research found that leptin and the receptor expressed in the NP cells.^35^ Moreover, leptin could promote the ECM catabolic metabolism of NP cells by MAPK and JAK2/STAT3 signaling and thus to promote IDD.^36^ Interestingly, leptin and the receptor also expressed in the AF cells and it could induce rat AF cells terminal differentiation via p38 MAPK and ERK1/2 MAPK signaling pathways.^37^ Besides, Han et al. reported the impact of leptin on disc cartilage endplate (CEP) in vitro and in vivo.^38^ Their findings showed that leptin increased the osteoblastic differentiation of CEP cells and promoted the disc endplate ossification by targeting MAPK/ERK signaling pathway. However, leptin is also a key appetite-regulating hormone, whose deficiency will cause polyphagia and lead to obesity and diabetes. A research by Natelson et al. found that obesity induced by deficiency of leptin receptor contributed to pathological changes and immature phenotype of the spinal disc.^39^ Besides, type II diabetes mellitus caused by leptin receptor knock out facilitated the disc cell apoptosis and ECM catabolism to promote IDD.^40^ Therefore, the roles of leptin signaling in IDD is complex and needs to be explored from multiple perspectives.

Visfatin, also termed as nicotinamide phosphoribosyltransferase (NAMPT), is another adipokine whose expression level is up-regulated in obesity, similar to leptin.^41,42^ As reported previously, visfatin could promote the IL-6 expression and ECM degradation of NP cells by JNK/ERK/p38-MAPK signaling to contribute to IDD.^43^ The NLRP3 inflammasome exerted important functions in IDD, and visfatin was also found to activate NLRP3 inflammasome to facilitate ECM degradation of rat NP cells through the MAPK/NF-κB signaling.^44^

Resistin is an important adipokine which might also function as a link between obesity and degenerative musculoskeletal diseases. Shin et al. revealed that resistin could promote obesity-related inflammatory IDD by MAPK and NF-κB signaling pathways.^45^ Another study also validated the functions of resistin in promoting the ECM degradation in rat disc NP cells.^46^ Additionally, it has been also reported that resistin could induce chemokine ligand 4 (CCL4) expression by p38-MAPK and NF-κB signaling in rat NP cells, leading to macrophage infiltration to promote IDD.^47^

Chemerin is another adipokine and is also termed as tazarotene-induced gene 2 (TIG2) or retinoid acid receptor responder 2 (RARRES2). A previous study reported that chemerin could increase NP cells inflammation, ECM degradation, and cell senescence by TLR4 and CMKLR1, as well as NF-kB signaling pathway.^48^ These findings might provide a new perspective to the causative roles of obesity in IDD.

Adiponectin is another dysregulated adipokine in obesity, widely involved in a diversity of pathological processes, including degenerative disc diseases.^49^ Yuan and colleagues found that adiponectin expression level was decreased in degenerated disks and NP cells.^50^ Furthermore, adiponectin could inhibit the TNF-α expression of degenerated NP cells to prevent the disc from degeneration. More recently, Ohnishi et al. found that AdipoRon, an agonist of adiponectin receptor, protected the IVD from degeneration in vitro and in vivo.^51^ In mechanism, AdipoRon reduced inflammation and ECM catabolism by activating the AMPK signaling and suppressed NF-κB signaling in IDD. These studies demonstrated that adiponectin was important for the disc homeostasis and provided a promising therapeutic target for IDD.

Progranulin (PGRN) is encoded by the progranulin (GRN) gene and is recently re-discovered as an adipokine.^52^ Interestingly, progranulin knockout could accelerate the disc matrix degeneration, potentially due to NF-κB and β-catenin signaling activation.^53^ Wang et al. study reported that progranulin could inhibit inflammation to protect IVD from degeneration by increasing IL-10 as well as decreasing IL-17.^54^ Taken together, these studies revealed that progranulin played important roles in the disc homeostasis, and might serve as a potential diagnosis biomarker and treatment target.

Omentin-1, referred to as intelectin-1, is also a critical adipokine with anti-inflammatory potential. Recently, Cabral et al. reported that mentin-1 facilitated human NP cell proliferation, and inhibited IL-1β-induced NP cell degradation, primarily by activating PI3K/Akt signaling pathway.^55^ Additionally, omentin-1 was also found to alleviate interleukin-1β-meditated human NP cells senescence in a Sirt1-dependent manner and thus to protect against disc degeneration.^56^ The above studies suggested that omentin-1 exerted critical functions in mitigating IDD and showed the potential in the target therapy for IDD.

In summary, obesity related adipokines have played important and complex roles in IDD pathogenesis and progression. A list of the effects and mechanisms of important adipokines functioned in IDD is presented in Table 1.

**Table 1:** Mechanisms of adipokines functioned in intervertebral disc degeneration

Adipokines	Experimental models	Signaling pathways	Functional mechanisms	References
Leptin	IDD in vitro (rat NP cells)	MAPK and JAK2/STAT3	Promote ECM degradation	Miao et al.^36^
Leptin	IDD in vitro (rat AF cells)	P38 and ERK1/2 MAPK	Promote cell terminal differentiation	Ding et al.^37^
Leptin	IDD in vitro and in vivo (rat CEP cells and rat lumbar disc)	MAPK-ERK	Promote cell osteoblastic differentiation and endplate ossification	Han et al.^38^
Visfatin	IDD in vitro and in vivo (human NP cells and rat anterior disc)	JNK/ERK/p38-MAPK	Promote inflammation and ECM degradation	Cui et al.^43^
Visfatin	IDD in vitro (rat NP cells)	MAPK/NF-κB	Promote inflammation and ECM degradation	Huang et al.^44^
Resistin	IDD in vitro (human NP cells)	MAPK/NF-κB	Promote inflammation and ECM degradation	Shin et al.^45^
Resistin	IDD in vitro (rat NP cells)	p38-MAPK	Promote ECM degradation	Liu et al.^46^
Resistin	IDD in vitro (rat NP cells)	p38-MAPK and NF-κB	Promote macrophages infiltration	Li et al.^47^
Chemerin	IDD in vitro (human NP cells)	NF-kB	Promote inflammation, ECM degradation, and cell senescence	Hu et al.^48^
Adiponectin	IDD in vitro (human NP cells)	N/A	Inhibit inflammation	Yuan et al.^50^
Adiponectin	IDD in vitro and in vivo (human NP cells and rat tail disc)	AMPK and NF-κB	Inhibit inflammation and ECM degradation	Ohnishi et al.^51^
Progranulin	PGRN knockout mice, and human peripheral blood mononuclear cells	N/A	Inhibit inflammation	Wang et al.^54^
Omentin-1	IDD in vitro (human NP cells)	PI3K/Akt	Promote cell proliferation, inhibit inflammation, apoptosis, and ECM degradation	Cabral et al.^55^
Omentin-1	IDD in vitro (human NP cells)	Sirt1	Inhibit cell senescence and ECM degradation	Huang et al.^56^

### Lipid metabolism disorder in IDD

Previous studies have shown that many obese patients have lipid metabolism disorders resulting in dyslipidemia, characterized by increased levels of low-density lipoprotein (LDL), free fatty acids, triglycerides (TG), and deceased levels of high-density lipoprotein (HDL).^17^ Obesity-meditated lipid metabolism disorders might be closely involved the IDD process. Firstly, the oxidized low-density lipoprotein (ox-LDL) is a harmful lipoprotein to the human body, which interacts with lectin-like ox-LDL receptor-1 (LOX1) to exert important functions. A previous research has reported that ox-LDL and LOX-l both expressed in the IVDs, and they were positively associated with the degree of IDD in the NP, EP cartilage, and outer annulus fibrous.^57^ In mechanism, oxLDL could significantly inhibit the NP cell viability and enhance LOX-1 and MMP-3 expression levels of NP cells by targeting NF-κB signaling. Besides, the increased fatty acids caused by obesity might have significant effects on the cell metabolism and function in degenerative disc diseases. Another research has revealed the association between IDD and the fatty acid. It was found that a stimulation of high levels of the fatty acid palmitic acid markedly increased the rat disc NP cells apoptosis rate and ECM catabolism, by targeting the ERK/MAPK signaling pathway.^58^ Additionally, the elevated levels of triglycerides and cholesterol caused by obesity might also play an important part in disc degeneration. Elevated triglycerides and cholesterol are primary risk factors for atherosclerosis, which negatively affected the blood supply and nutrient delivery to the already poorly vascularized disc, contributing to IDD progression.^59^ In summary, lipid metabolism disorder is deeply involved in IDD pathophysiology, which might provide promising therapeutic targets for IDD treatment.

### Obesity related inflammatory cytokines in IDD

**Fig. 1:**
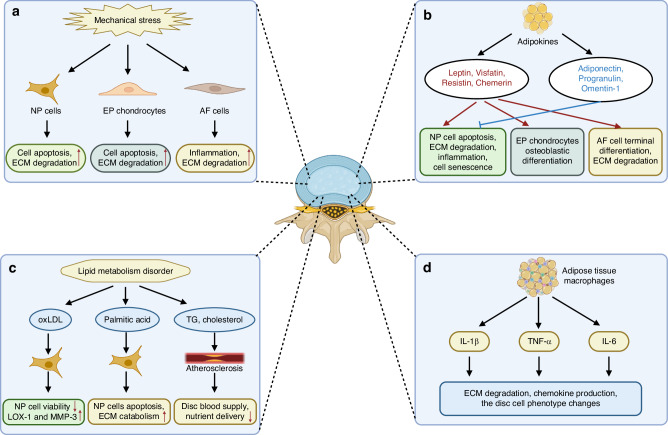
Schematic diagram of the mechanisms of obesity-related factors in intervertebral disc degeneration. **a** Mechanical stress contributes to IDD by regulating NP cell apoptosis and ECM degradation, EP chondrocyte degeneration, AF inflammation and ECM degradation. **b** Obesity related adipokines have played complex roles in IDD. Several adipokines including leptin, resistin, visfatin and chemerin exert damaging effects on the disc cells, while adiponectin, progranulin and omentin-1 exert protective effects on the disc cells. **c** Lipid metabolism disorder contributes to IDD. The oxLDL could inhibit the viability of NP cells and enhance LOX-1 and MMP-3 expression levels of NP cells. The fatty acid palmitic acid increases the disc NP cells apoptosis and ECM catabolism. Elevated triglycerides and cholesterol could decrease the blood supply and nutrient delivery to the disc. **d** The adipose tissue macrophages secrete various inflammatory cytokines, including IL-1β, TNF-α, and IL-6 to promote IDD progression

The adipose tissue is considered as a very complex organ system, with macrophages constituting an important source of inflammatory cytokines. In lean adipose tissue, there exists a homeostasis between adipocytes and resident immune cells. Importantly, the Treg cells can produce several anti-inflammatory cytokines (e.g. IL-4 and IL-10), which augment M2 macrophage phenotype.^60^ During obesity, the adipose tissue macrophages (ATMs) undergo a phenotypic shift from M2 state (anti-inflammatory) to M1 state (pro-inflammatory), with a property of enhanced proinflammatory cytokines release, including TNF-α, IL-1β and IL-6.^61^ Obesity-meditated chronic inflammation has been deeply involved in the occurrence of obesity-related diseases, including intervertebral disc degeneration.^60^ Evidence has shown that those secreted inflammatory cytokines (e.g. TNF-α, IL-1β, IL-6 and IL-17) could facilitate ECM degradation, chemokine production and the disc cell phenotype changes, and thus to contribute to IDD progression.^62^ Particularly, TNF-α and IL-1β are two commonly studied inflammatory cytokines. In mechanism, TNF-α and IL-1β were found to promote catabolism and inhibit anabolism of extracellular matrix of the disc.^60,63^ Besides, TNF-α and IL-1β could also promote some pro-inflammatory mediator expressions in NP, including IL-6, IL-8, IL-17, iNOS, etc.^60^ These findings suggested that obesity related pro-inflammatory cytokines might serve as critical links between obesity and IDD (Fig. 1).

## Obesity and OSL

Ossification of spinal ligaments (OSL) is a degenerative spinal disease represented by ossification of the ligamentum flavum (OLF) and posterior longitudinal ligament (OPLL), leading to stenosis of spinal canal and intervertebral foramen, compression and injury to spinal cord and nerve roots. The severity of injury is correlated with the degree of stenosis. Patients may present with a series of symptoms, including sensory disturbances, motor impairments, and autonomic dysfunction. In recent years, the correlation between ossification of spinal ligaments and obesity has become increasingly evident, with obesity emerging as a prevalent exacerbating factor for diffuse ossification of spinal ligaments, even rendering the entire ligamentous structure susceptible to ossification.^64–66^ Metabolic disorders associated with ossification of spinal ligaments may impact the bone metabolism and lead to ectopic ossification. However, further study is needed on the effects of mechanical stress caused by obesity on OSL currently.

Several epidemiological research has validated the relationship between OLF and obesity. A cross-sectional research from Japan included 458 patients demonstrated the association between dyslipidemia and the incidence of OLF exhibited a stronger correlation compared to other risk factors. They proposed that the progression of OLF lesions might be exacerbated by lipid metabolic abnormalities associated with visceral adiposity.^67^ The accumulation of visceral fat appears to better explain the pathogenic factors of OLF than a mere increase in BMI. A further retrospective analysis has validated the independent correlation between OLF and obesity, with the impact of other related factors (e.g. gender) being distinct, implying that degenerative changes might not hold the same importance in OLF as other obesity-related factors.^64^ Our research team has also conducted relevant studies and confirmed that high BMI was significantly positively correlated with the onset and severity of OLF, and obesity served as an independent risk factor for OLF.^68,69^ Regarding OPLL, the latest epidemiological research indicated that obese patients were more prone to cervical OPLL, and the exacerbation of cervical OPLL was associated with the degree of obesity.^65,70^ It is worth mentioning that spinal epidural lipomatosis, characterized by excessive adipose tissue in epidural space leading to spinal canal stenosis, is closely associated with obesity. Research has revealed that the levels of inflammatory cytokines in obese spinal epidural lipomatosis patients were obviously elevated, suggesting that obesity-induced chronic inflammation might play important roles.^71^ While the correlation between obesity and OSL has been established, the precise molecular mechanisms underlying this relationship remain unclear. Subsequent sections will delve into elucidating the mechanisms by which obesity factors facilitate OSL.

### Adipokines function in OLF progression

Adipokines, including leptin, lipocalin, resistin, and visfatin, are predominantly secreted from white adipose tissue (WAT). Adipokines as well as the receptors have been found in adipose tissue, articular cartilage, and spinal bone marrow. Obesity and hyperlipidemia are linked to lipid metabolism disorders and dysfunction of adipose tissue, rendering adipose tissue susceptible to immune infiltration and change into a pattern of adipokine secretion.^60^ Adipokines promote the progression of OLF by stimulating inflammatory reactions and promoting pathological changes in the degeneration of the ligamentum flavum.

Leptin is a common adipokine which can be released from WATs, bones, and joints.^35,72,73^ It is positively correlated with body fat mass^74^ and can regulate the differentiation of several cell lines.^75,76^ An experiment in Japan revealed a significant positive association between leptin/BMI ratio and serum insulin expression as well as the vertebrae number affected by ligamentous ossification.^77^ According to reports, leptin can activate PI3K/Akt, JAK/STAT, and MEK/ERK signaling. Additionally, it has a protective effect by inducing the expression of cyclin D3 and promoting proliferation of human marrow cells.^78^ Nevertheless, leptin exerts important function in inflammation and cellular metabolism, which cannot be underestimated. Excessive adipose tissue leads to hyperleptinemia and systemic inflammatory responses. Interestingly, Fan et al. found that leptin markedly up-regulated alkaline phosphatase (ALP) and osteocalcin mRNA of the ligamentum flavum (LF) cells, and its effects were dose and time-dependent.^10^ The above discoveries showed that LF cells are quite sensitive to leptin, and leptin may promote osteogenic differentiation of LF cells. Hence, gaining a deeper understanding of the roles of leptin in OLF is paramount.

Resistin is found in many tissues including the lumbar and skeletal muscles.^79,80^ OLF represents a form of heterotopic ossification, characterized by endochondral ossification akin to the development of long bones, mediated by osteoblasts and mineralizing into mature, histologically normal bone.^81^ Although the influence of resistin on OLF is not well comprehended, existing research has confirmed that resistin is highly expressed in mesenchymal stem cells (MSCs) undergoing osteogenic differentiation, and it can promote the progression of osteogenic differentiation in MSCs via PI3K/AKT/mTOR pathway.^82^ Hence, resistin may emerge as a potential adipokine promoting OLF.

Lipocalin is an adipokine involved in systemic glucose homeostasis and obesity, produced by adipose tissue, and secreted at lower levels in skeletal muscle and venous tissue.^83^ Interestingly, it is proposed that Lipocalin-2 (LCN2) should be more appropriately termed as osteokine because its expression level in bone is significantly higher than in WAT.^84^ Meanwhile, the regulation of bone metabolism by LCN2 may also be related to FGF23.^85^ FGF23 can facilitate the activation of cAMP signaling in bone cells, resulting in the overproduction of LCN2 in the kidneys.^86^ Simultaneously, overexpression of LCN2 stimulates the production of RANKL and IL-6, leading to decreased osteoblast differentiation.^87^ However, the exact role of LCN2 in OLF remains unclear. As a potential target against OLF, its specific mechanism warrants further detailed investigation.

Visfatin (NAMPT) modulates the pathogenesis of obesity-related diseases through pathways involving oxidative stress, apoptosis, inflammation, lipid metabolism, etc.^88^ NAMPT gradually increases during osteogenic differentiation of BMSCs and may serve as a marker of osteoblast differentiation.^89^ Li et al. reported that NAMPT knockdown or using the inhibitor FK866 could result in reduced intracellular NAD concentration and diminished osteogenic capacity.^90^ Ling et al. reported that osteogenic differentiation of BMSCs is reduced in NAMPT-deficient mice, while knockdown of NAMPT can attenuate the increase in RUNX2 promoter acetylation associated with osteogenic differentiation.^91^ Further studies have shown that in the ossified ligamentum flavum tissues, NAMPT is up-regulated, while the miRNA miR-182 involved in cell differentiation is downregulated.^92^ Additionally, miR-182 can suppress OLF by downregulating NAMPT.^93^ However, research on the association between NAMPT and OLF is quite limited, and further elucidation of the detailed information concerning their relationship is required. The list of the mechanisms of adipokines functioned in OLF is presented in Table 2.

**Table 2:** Mechanisms of adipokines functioned in ossification of the spinal ligaments

Adipokines	Experimental models	Signaling pathways	Functional mechanisms	References
Leptin	OLF in vitro (human LF cells)	STAT3, JNK and ERK1/2	Promote osteogenic differentiation of thoracic ligament flavum cells	Fan et al.^10^
Visfatin	OLF in vitro (human LF cells)	MiR-182-NAMPT	Promote ossification of ligamentum flavum	Zhang et al.^93^
Leptin	OPLL in vitro (human PLL cells)	ERK1/2, p38 MAPK, and JNK	Promote osteogenic differentiation of posterior longitudinal ligament cells	Feng et al.^138^

### Dyslipidemia accelerates the progression of OLF

Lipid metabolism has been reported to regulate osteoblasts by targeting Wnt signaling and is intricately linked to bone metabolism and calcification processes.^94–96^ Epidemiological research has validated the relationship between dyslipidemia and the development of OLF, with a stronger correlation between dyslipidemia and OLF incidence compared to other risk factors.^67^

Cholesterol was reported to affect the activity of Indian hedgehog protein (IHH), thereby regulating the development of articular cartilage, highlighting the significant role of cholesterol in cartilage development.^97^ Numerous studies indicated that enhancing cholesterol efflux or cholesterol metabolism might aid in shielding chondrocytes from the impact of inflammation.^98–100^ Parhami et al. discovered that cholesterol targeted and inhibited HMG-CoA reductase, which led to reduced activity and expression of the osteogenic gene ALP, consequently repressing bone mineralization.^101^ Sheng et al. further revealed that cholesterol can selectively activate canonical Wnt signaling by targeting Disheveled (Dvl), the membrane receptor of the Wnt signaling, to promote osteogenic activity.^102^ Li et al. reported that cholesterol had dual effects on osteogenic differentiation of BMSCs and endogenous cholesterol were necessary for BMSCs osteogenic differentiation.^103^ These studies showed that the impact of cholesterol on OLF might depend on cell type, cholesterol type, and cholesterol concentration.

Elevated ox-LDL levels represent a hallmark of obesity-associated dyslipidemia. Low-density lipoprotein (LDL) can undergo non-enzymatic oxidation to become oxidized low-density lipoprotein (ox-LDL), the active form of LDL. Ox-LDL binds to the receptor LOX1, reducing PG synthesis within the cartilage matrix, increasing intracellular reactive oxygen species generation, and activating NF-κB. This cascade further stimulates the production of pro-inflammatory cytokines, chemokines, enzymes, as well as adhesion molecules.^104^ Research has revealed that ox-LDL markedly promoted calcium deposition and enhanced ALP and OCN expression in endothelial progenitor cells, indicating that ox-LDL might facilitate ectopic ossification.^105^ However, another report indicated that oxLDL could disrupt Wnt signaling in a CD36-dependent manner, thereby inhibiting the differentiation of MSCs into osteoblasts.^106^ Hence, the roles of ox-LDL in the ectopic ossification of ligamentum flavum cells necessitate further comprehensive investigation.

Lipoprotein receptor-related protein (LRP), identified as a cell surface protein recently, functions as an endocytic receptor. Studies have revealed that LRP5 or LRP6 could interact with WNT to stabilize β-catenin, which triggers the β-catenin target genes expression, ultimately promoting osteoblasts to differentiate into mature osteocytes.^107^ Experiments by Riddle and colleagues further uncovered a distinct skeletal function of Wnt-LRP5, namely its ability to enable osteoblasts to oxidize fatty acids.^108^ Wnt can also be modulated by fatty acids, with a cis-unsaturated fatty acyl group covalently attached to a conserved serine residue and palmitoylation occurring on a conserved cysteine residue, regulating Wnt protein secretion. The modification is crucial for Wnt activity, revealing that specific lipid modifications are necessary for the proper intracellular transport of Wnt proteins during secretion.^109,110^ Although the function of fatty acids as energy source in Wnt- mediated osteoblast differentiation has been investigated, its amount used to produce ATP in bone cells remains unclear.^111^

Elevation of circulating free fatty acids (FFAs) is also a hallmark of obesity-associated dyslipidemia. The primary cause of elevated FFAs release into the circulation from adipose tissue is the impaired inhibition of lipolysis in adipocytes.^112^ FFAs can promote macrophage inflammatory responses via toll-like receptors (TLRs) and downstream signaling pathways involving the phosphorylation of TAK1, JNK, p-38, c-Jun as well as NF-κB, resulting in production of cytokines, iNOS, and COX-2.^113^ They can also induce infiltration and activation of macrophages in adipose tissue, thus promoting the increase of TNF-α and IL-6, which can promote the occurrence and progression of ligamentum flavum ossification.^114,115^ In vitro experiments reported that long-chain saturated fatty acid palmitate could inhibit RUNX2 and osteocalcin expression in fetal rat calvarial cells.^116^ Peroxisomes can also metabolize long-chain fatty acids, and the number of peroxisomes increases during the differentiation process of osteoblasts, indicating that more fatty acids are utilized as substrates for energy supply.^117^ Besides osteoblasts, fatty acids and their derivatives can also act as signaling molecules, binding to receptors on various joint tissue cells, such as chondrocytes, osteoclasts, and synovial cells.^118^

### Inflammatory factors promote the development of OLF

Mature adipocytes possess the capacity to synthesize pro-inflammatory proteins, thereby eliciting infiltration and activation of macrophages in adipose tissue, with macrophages constituting a significant source of inflammatory cytokines in the body.^119^ Inflammatory factors have been confirmed as important pathogenic factors in OLF. In a retrospective study,^120^ results suggested that a high systemic immune validation index was a critical risk factor for OLF. Various inflammatory factors, including interleukins, TNF-α, and TGF-β may play crucial roles in OLF progression through different downstream pathways. A recent research demonstrated that TNF-α, IL-6 and leptin secreted by epidural fat were obviously up-regulated in OLF than control.^121^ However, whether epidural fat directly affects the OLF process needs to be further explored.

Inflammatory cytokines might play pivotal roles in hypertrophy or ossification of LF. During LF ossification process, LF cells cultured with IL-1α, IL-6, PGE2 and TNF-α, exhibited markedly increased synthesis of DNA and mRNA of type I, V, XI collagen, and osteocalcin.^122^ Another research reported that IL-6 derived from M1 macrophages could facilitate LF cells osteogenic differentiation.^123^ Subsequent research has revealed that IL-6 could increase BMP2, RUNX2, OSX, OCN, and ALP expression through the p38 MAPK pathway.^124^ However, IL-6 can stimulate osteoclasts and inflammation through the gp130 signaling pathway, thereby inhibiting bone formation, exerting profound impacts on bone metabolism.^125^ Therefore, a deeper exploration of the mechanism between IL-6 and OLF is warranted.

IL-17 is an important cytokines family mainly synthesized and secreted by CD4^+^ helper T cells 17 (Th17). The function of IL-17 in osteoblast formation and osteogenic activity remains undetermined. On one hand, IL-17A expression rapidly increases at the early site of fractures, contributing to bone healing. However, IL-17A gene knockout mice show reduced proliferation of osteoblast precursors and osteoblast differentiation, impacting the process of fracture healing.^126,127^ On the other hand, Kim et al. found that IL-17 can inhibit osteoblast differentiation in rat calvarial cells.^128^ Additionally, Shaw et al.^129^ recently found that IL-17A stimulates osteoblast expression of the Wnt inhibitor sFRP1, thereby inhibiting osteoblast differentiation and bone mineralization, and neutralizing antibodies to sFRP1 could block this effect of IL-17A. Our recent research has found elevated IL-17A levels in OLF tissue, where IL-17A promoted proliferation and osteogenic differentiation of LF cells by modulating the β-catenin signaling pathway.^130^ Hence, the osteogenic role of IL-17 is intricate, and further investigation into the impact of IL-17 on osteoblast differentiation and OLF holds significant importance for elucidating the inflammatory mechanism underlying OLF ossification.

TNF-α is an essential cytokine and participates in the pathogenesis of numerous human diseases.^131^ The transgenic animal models have demonstrated the significant effects of TNF-α on bone fracture healing.^132^ Interestingly, Zhang et al.‘s experiment revealed that TNF-α protein levels were elevated in OLF, and could increase osteogenic cell differentiation-related genes expression.^133^ Similarly, Wang et al. verified that serum TNF-α level in TOLF patients was markedly elevated than control, and TNF-α could induce the expression of Osterix and its downstream target genes OCN as well as ALP.^134^ However, some studies reported that TNF-α could reduce SOX9 expression, which is important for cell osteogenic differentiation.^135,136^ Therefore, the function of TNF-α is complex, and further research on TNF-α might be instrumental in elucidating the specific mechanism of OLF.

### Obesity and OPLL

Similar to OLF, OPLL is also closely associated with obesity. A recent cross-sectional research from Japan involving 92 OPLL patients and 246 control subjects discovered a correlation between dyslipidemia and the incidence of OPLL, and the study also noted that OPLL patients had higher BMI compared to control, suggesting that visceral adiposity and elevated BMI were implicated in the pathogenesis and progression of OPLL.^137^ Leptin, a well-known adipokine, also contributes to OPLL. Feng et al. reported that leptin stimulation obviously facilitated osteogenic differentiation of PLL cells by ERK1/2, p38 MAPK, as well as JNK signaling pathways.^138^ Another study showed that co-stimulation of leptin and mechanical stress enhanced OPLL via the MAPK, JAK2-STAT3, as well as PI3K/Akt pathways.^139^ A comparative research about serum and tissue cytokines in OPLL patients revealed that leptin expression was elevated in serum compared to tissue, substantiating the notion that leptin could serve as a biomarker for the disease.^140^ Despite the preventive effects of n-3 polyunsaturated fatty acids (PUFA) on ectopic ossification,^141^ a study assessing the association between FAs and the incidence of OPLL revealed no association between n-3 PUFA and risk of OPLL.^142^ Inflammatory factors elevated due to obesity similarly contribute to the formation of OPLL. In an in vitro cell model of OPLL, TNF-α was not only expressed in degenerated and ossified ligament tissues but also enhanced ALP activity and collagen synthesis in posterior longitudinal ligament cells, suggesting the important function of TNF-α in OPLL pathogenesis.^143^ IL-6 is capable of promoting the osteogenic differentiation of MSCs. Researchers exploring the downstream mechanisms of IL-6 in promoting OPLL discovered that IL-6 could activate STAT3 to induce the inhibitory effect of BMPER, a target gene of miR-135b, and thus to inhibit the osteogenic differentiation induced by cyclic tensile strain.^144^

**Fig. 2:**
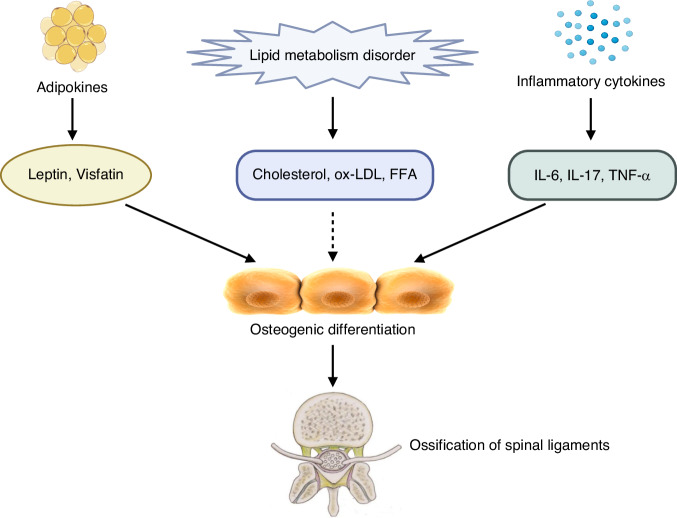
Schematic diagram of the mechanisms of obesity functioned in ossification of the spinal ligaments. Several adipokines, including leptin and visfatin, could promote the osteogenic differentiation of ligamentum flavum cells or posterior longitudinal ligament cells, to facilitate ossification of ligamentum flavum or posterior longitudinal ligament. Lipid metabolism disorder might potentially contribute to ossification of the spinal ligaments via cholesterol, ox-LDL and FFA. Inflammatory cytokines, including IL-6, IL-17, and TNF-α enhance the osteogenic differentiation of ligamentum flavum cells to promote the development of ossification of ligamentum flavum

In summary, obesity can advance OSL progression through factors like adipokines, dyslipidemia, and inflammatory cytokines (Fig. 2). While OSL represents a multisystem, multifactorial condition, weight loss may emerge as a straightforward and efficacious treatment avenue for preventing or mitigating OSL progression in the future, with the associated molecular or cellular mechanisms awaiting deeper exploration.

## Obesity and OA

OA is a clinical syndrome marked by joint pain, varying levels of functional impairment, and diminished quality of life. OA first presents as molecular disorder, then progresses to anatomical and/or physiological disruptions, ultimately culminating in the disease.^145^ The etiology of OA is still unclear, yet cross-sectional and longitudinal research consistently demonstrate a correlation between obesity (typically assessed via BMI) and knee OA prevalence and incidence. Obesity is recognized as a primary modifiable risk factor.^146–150^ An early finding revealed that individuals with a BMI exceeding 30 kg/m² have a 6.8-fold increased risk of knee arthritis compared with those with a normal weight.^151^ Furthermore, researchers have indicated that the lifetime risk of developing symptomatic knee arthritis is higher in individuals with a BMI ≥ 30 kg/m² compared to their non-obese counterparts (19.7% vs. 10.9%).^152^ Obesity not only amplifies the load on weight-bearing joints (e.g. knee) but also contributes to joint misalignment and unfavorable joint mechanics, thereby accelerating cartilage degradation and ultimately resulting OA.^15^ Inflammation is also pivotal in the progression of osteoarthritis, which can be originated from adipose tissue within joint cavity.^153^ Besides, when the lipid metabolism homeostasis is disturbed, it predisposes the body to disease like OA.^154^ Furthermore, extensive research indicates that individuals with disrupted lipid metabolism have an elevated risk of OA.^155^ In obese patients, adipose tissue generates adipokines like leptin, adiponectin, resistin, visfatin, as well as inflammatory cytokines such as TNF-α, IL-1, and IL-6.^156–158^ These factors are intricately linked with obesity and can profoundly impact the onset and advancement of OA.

### Mechanical stress promotes the development of OA

Excess body weight not only augments loading on weight-bearing joints of the lower extremities, but also induces detrimental joint mechanics, potentially leading to joint dislocation, particularly in the knees, thus amplifying mechanical stress.^159^ Research has indicated a positive correlation between high body weight and load on major joints of the lower extremities.^160^ Furthermore, obesity contributes to a decline in muscle strength that is critical to maintain joint stability, thus impairing the joint’s capacity to endure mechanical stress.^161^ Mechanical stress can further modify inflammatory status of chondrocytes. High-intensity cyclic tensile strain applied to chondrocytes can markedly elevate inflammatory mediators via signaling pathways including FAK, ERK, JNK, p38, and NF-κb.^162–165^ Mechanical stress can lead to a down-regulation of the FBXW7, which in turn accelerates the senescence of cultured chondrocytes and murine articular cartilage.^166^ Besides cartilage, mechanical stress also adversely affects subchondral bone. Mechanical overload can result in thickening of subchondral cortical bone, and excessive stress in later stages can promote cartilage degeneration in OA, worsening the condition.^167,168^ Under simulated abnormal mechanical loading in cartilage, the pro-inflammatory environment and the reduced OPG/RANKL ratio lead to chondrocyte apoptosis and cartilage matrix degradation through matrix metalloproteinases (MMPs).^169^ Although excessive joint mechanical load is a crucial factor in obesity-mediated OA, the altered biomechanics cannot entirely explain the elevated OA risk in non-weight-bearing joints like the hands and wrists of obese individuals. This indicates that the risk of OA has systemic and non-mechanical influences.^170^ Since OA is a multi-systemic and multifactorial disease, the increased mechanical stress resulting from obesity represents only one facet of its risk factors.

### Adipokines function in the development of OA

The previously mentioned adipokines are secreted not only by WAT, but also by other joint tissues including cartilage, synovium, osteophytes, and meniscus, especially in the joint tissues of OA patients. Furthermore, adipokines seem to not directly impact osteophyte formation in OA but rather influence the pro-inflammatory environment of OA joints.^171^ Dyslipidemia in obese individuals can prompt the secretion of numerous adipokines, which can hasten the late-stage cartilage degeneration process in OA through synergistic action with cartilage matrix-degrading enzymes like MMPs. These adipocyte-derived factors also act as pro-inflammatory agents involved in the inflammatory response process of osteoarthritic joints, closely linked with the pathological process of OA.^172^

Leptin has a catabolic function in chondrocyte proliferation, promoting the secretion of critical cartilage-degrading mediators (e.g. TNF-α, IL-1β, and IL-6).^173^ Compared to healthy subjects, systemic leptin levels are elevated in OA patients.^174^ Several studies have found that leptin were significantly up-regulated in OA and positively correlated with the severity of the disease and pain levels.^175–177^ In terms of mechanism, leptin could promote matrix degradation metabolism via JAK2/STAT3 and MAPK pathways.^36^ Furthermore, leptin could activate RhoA/ROCK signaling and induce cell cytoskeletal remodeling.^114,178^ Additionally, leptin could promote STAT3 expression, subsequently inhibiting REDD1 expression and consequent p70S6K phosphorylation, thereby activating mTORC1 and suppressing chondrocyte autophagy in the progression of OA.^179^ Leptin also impacts subchondral bone dysfunction. The abnormal increase in leptin in OA subchondral bone osteoblasts and the heightened leptin synthesis can induce abnormal expression of TGF-β1, osteocalcin, and ALP in osteoblasts, thereby causing dysfunction in subchondral bone osteoblasts in OA, fostering osteophyte formation.^180^

As the adipokine with the highest concentration in peripheral blood, adiponectin has been discovered to express in synovial cells, infrapatellar fat pad, osteophytes, cartilage, and bone tissue.^181^ Many studies have suggested that circulating adiponectin level was up-regulated in OA compared to healthy controls.^182–184^ Importantly, research has indicated a stronger correlation between adiponectin, rather than leptin, and the clinical severity of knee OA in females, highlighting its clinical relevance in the pathogenesis of OA.^185^ Adiponectin induces inflammatory responses by stimulating chondrocytes to produce interleukin-6, interleukin-8, MMP-1, etc.^186^ Adiponectin can also enhance VCAM-1 expression in chondrocytes, thereby perpetuating the degradation process of inflammatory OA cartilage.^187^ Moreover, studies have revealed a close correlation between AdipoR1 and cartilage-specific components mRNA level, indicating adiponectin’s potential role in matrix remodeling.^188^ While research has suggested an involvement of adiponectin in the pathophysiology of OA, current studies have mainly explored its adverse effects on OA formation, leaving its potential specific protective role during the onset of OA uncertain.

It has been confirmed that resistin is associated with knee osteoarthritis.^175^ Epidemiological and clinical research reveal a positive association between serum and synovial fluid resistin levels and the severity, synovitis, and structural abnormalities of osteoarthritis patients.^189–191^ It is believed that resistin promotes the progression of OA by promoting synthesis of MMPs and pro-inflammatory cytokines in chondrocytes.^80^ Resistin was reported to suppress miR-381 expression to enhance the expression of VCAM-1 and monocyte adhesion in human osteoarthritis synovial fibroblasts (OASFs), promoting synovial inflammation.^192^ Furthermore, resistin can down-regulate type II collagen and proteoglycans expression and up-regulate ADAMTS-4, MMP-1, and MMP-3 expression in chondrocytes, accelerating cartilage degradation.^193^ Resistin can stimulate chondrocytes to generate inflammatory factors like IL-6, TNF-α, and prostaglandin E2, exacerbating joint inflammation.^194^

Visfatin can participate in OA pathogenesis by generating inflammatory mediators, and disrupting the expression of proteins required to maintain chondrocyte phenotype.^195–197^ Visfatin was reported to induce lymphocytes to generate IL-1β, IL-6, and TNF-α, exerting pro-inflammatory effects, which could also promote the progression of OA.^198^ Chemerin, another adipokine predominantly expressed in white adipose tissue, can interact with the ChemR23 receptor to induce the chemotaxis of immune cells to inflammatory sites. In OA, as a component of the inflammatory cascade, immune cells like macrophages are recruited to synovium.^199^ Human articular chondrocytes possess inherent capabilities to express chemerin and ChemR23 to facilitate inflammatory signal transduction,^200^ suggesting a potential role of chemerin in OA.

Omentin-1 is another adipokine and its expression level is negatively associated with various diseases progression, including diabetes, obesity, and osteoarthritis.^55,201^ Research has shown that omentin-1 can induce IL-4-dependent anti-inflammatory responses and M2 macrophage polarization in OA synovial fibroblasts to mitigate cartilage degradation.^202^ Hence, further research into its potential for in vivo treatment of OA is crucial. The list of vital adipokines in OA is on the Table 3.

**Table 3:** Mechanisms of adipokines functioned in osteoarthritis

Adipokines	Experimental models	Signaling pathways	Functional mechanisms	References
Leptin	OA in vitro and in vivo (human chondrocytes and rat osteoarthritis model)	STAT3/REDD1/mTORC1	Promote apoptosis and inhibit autophagy of chondrocytes	Huang et al.^179^
Leptin	OA in vitro (human osteoarthritis osteoblasts)	P38 and ERK1/2	Promote dysfunction in subchondral bone osteoblasts in OA and osteophyte formation	Mutabaruka et al.^180^
Adiponectin	OA in vitro (human OA chondrocytes)	AMPK and JUK	Promote matrix degradation in OA cartilage	Kang et al.^186^
Adiponectin	OA in vitro (human and mouse chondrocytes)	JAK2, PI3K and AMPK	Promote the degradation process of inflammatory OA cartilage	Conde et al.^187^
Adiponectin	OA in vitro (human OA chondrocytes)	N/A	Promote matrix remodeling during OA	Francin et al.^188^
Resistin	OA in vitro (human OA synovial fibroblasts)	PKCα, p38, and JNK	Promote synovial inflammation	Chen et al.^192^
Resistin	OA in vitro (human OA chondrocytes)	NF-κB and C/EBPβ	Promote cartilage degradation	Zhang et al.^193^
Visfatin	OA in vitro (human OA synovial fibroblasts)	ERK, p38 and JNK	Promote synovial inflammation	Wu et al.^195^
Visfatin	OA in vitro (murine chondrocytes and osteoblasts)	N/A	Promote inflammation response	Laiguillon et al.^197^
Omentin-1	OA in vitro (human OA synovial fibroblasts)	PI3K, ERK and AMPK	Mitigate inflammation response and M2 macrophage polarization	Ko et al.^202^

### Abnormal lipids promote OA

Changes in lipid metabolism might be involved in the pathophysiology of OA. Epidemiological research has revealed a positive association between hypercholesterolemia and OA, indicating cholesterol as a possible risk factor for OA.^203^ The decreased serum HDL-c levels observed in OA patients may be associated with the onset of OA. Animal experiments have shown that changes in the HDL-c metabolic pathway could negatively impact cartilage homeostasis, leading to osteoarthritis.^204^ A study in Iran investigating the cause of OA found that serum TG concentrations in OA were obviously elevated compared to control, suggesting that TG metabolic disorders might be one of the factors contributing to the onset of OA.^205^ Besides, another report identified a positive association between ox-LDL levels and OA severity.^206^

Rodent studies involving *ApoE*^*−*^^*/*^^*−*^ mice and diet-induced hypercholesterolemic rats have demonstrated that hypercholesterolemia could stimulate OA-like changes, emphasizing the function of cholesterol in OA.^207^ As previously mentioned, the impairment of cholesterol efflux genes in chondrocytes is accompanied with increased intracellular lipid levels, positively correlated with disease severity. Enhancing cholesterol efflux or metabolism might prevent chondrocytes from inflammatory damage. There is a report indicating that RORα plays a crucial role in regulating lipid metabolism.^208^ RORα is a downstream target in cholesterol metabolic pathways. RORα can up-regulate genes related to cartilage degradation and down-regulate anabolic factors.^209,210^ Additionally, RORα might regulate OA development via IL-6/STAT3 pathway.^211^ It’s evident that RORα plays a significant role in modulating the cholesterol metabolic pathway, potentially serving as a therapeutic target for metabolic OA.

Triglycerides are esters composed of glycerol and three fatty acids. Up-regulated serum triglyceride level is considered as a risk factor for OA.^212^ Physiologically, triglycerides in adipose tissue are gradually hydrolyzed into glycerol and FFAs by lipases. Fatty acids are categorized into saturated fatty acids (SFAs), monounsaturated fatty acids (MUFAs), and polyunsaturated fatty acids (PUFAs). As the level of SFAs increases in the diet of OA patients, the degree of reduction in joint space width also increases, revealing that SFAs might exacerbate structure damage in OA.^13^ Mechanistically, in the SFA-induced OA chondrocyte model, there is an upregulation of IL-1β and MMP-13 mRNA expression, accompanied by a decrease of Col-2 and SOX9 levels, and reduced glucose uptake by chondrocytes.^213^ Studies on the association between MUFAs and OA progression are relatively scarce. A previous research reported a higher levels of synovial fluid MUFAs in OA compared with control.^214^ In the presence of TNF-α induction, MUFAs could suppress PTGS2 and MMP-1 mRNA expression, thereby inhibiting cartilage degradation.^215^

According to the position of double bonds, PUFAs can be classified into omega-3 (n-3) and omega-6 (n-6) PUFAs, and the balance between these molecules plays a crucial role in modulating gene expression in the body. A recent report suggested that plasma levels of n-6 PUFA in male OA patients were positively associated with the levels of joint effusion and knee joint structural damage.^216^ Researchers gathered plasma samples from 167 knee osteoarthritis patients and discovered that those with a higher n-6/n-3 PUFA ratio exhibited lower pain thresholds and more pronounced restrictions in joint movement and activity.^217^ Furthermore, n-3 PUFAs up-regulation and n-6 PUFAs down-regulation were reported to mitigate OA progression.^218^ Another study discovered that when incubated with bovine chondrocytes, n-3 PUFAs could suppress cartilage degradation-related genes expression.^219^ Besides, n-6 PUFAs could promote IL-6 secretion in chondrocytes.^220^ Moreover, n-6 PUFAs showed positive correlations with obesity-related OA and inflammatory adipokines.^221^ Hence, it’s conceivable that up-regulating n-3 PUFAs and down-regulating n-6 PUFAs in the body might be a strategy to decelerate OA.

In osteoarthritis, inflammation widens vascular pores, hastening the influx of oxidatively modified ox-LDL from the joint exterior into the synovial fluid.^222^ Ox-LDL binds to its clearance receptor LOX1, reducing cell viability and PG synthesis in the cartilage matrix, increasing intracellular reactive oxygen species production, consequently activating NF-κB.^223^ Compared to control, there’s an elevation in chondrocyte LOX-1 expression and localization in OA cartilage.^224^ Furthermore, ox-LDL/LOX-1 can induce the production of VEGF in chondrocytes, contributing to cartilage degradation.^225^ Ox-LDL can also promote the production of inflammatory cytokines and MMPs via activating synovial macrophages, endothelial cells, as well as synovial fibroblasts.^226^

### Inflammatory cytokines promote the development of OA

At molecular level, OA is characterized by the presence of numerous inflammatory factors, including cytokines and chemokines, all integral to the innate immune response to joint damage.^227^ In the synovial fluid and tissues of OA, an increasing array of pro-inflammatory mediators has been observed, including IL-1, TNF-α, IL-6, MCP-1, IP-10, and MIG.^228^

Macrophages constitute the primary infiltrating inflammatory cells in OA synovial fluid. Studies employing flow cytometry to examine synovial infiltration have consistently identified CD4^+^ macrophages as the most abundant inflammatory cells, followed by CD4^+^ T lymphocytes.^229^ Evidence from experiments of obese rats induced by high-carbohydrate/high-fat diets also indicates spontaneous infiltration of inflammatory M1 macrophages into joint synovium, exacerbating OA-like pathological changes.^230^ The inflammatory cytokines released by M1 macrophages can drive the damage process of chondrocytes through NF-κB, TGF-β, JNK, p38 MAPK, and β-catenin signaling.^231^ These cytokines can promote osteoclast differentiation and bone resorption, decrease type II collagen expression, and up-regulate multiple matrix-degrading proteases.^232,233^

IL-1β is a principal pro-inflammatory cytokine and is up-regulated in OA synovial fluid, synovium, subchondral bone, as well as cartilage. IL-1β activates cells by interacting with the receptor IL-1RI, which is heightened in OA chondrocytes and synovial fibroblasts compared to normal cells.^234^ IL-1β could decrease type II collagen and the aggregating protein aggrecan expression in chondrocytes.^235–238^ By using sodium iodoacetate-injected IL-1 receptor antagonist deficient mice, researchers found that IL-1 receptor antagonist deficiency not only accelerated cartilage destruction in the mouse model of osteoarthritis, but also increased levels of chondro-inflammatory factors.^239^ Interestingly, a novel intra-articularly injected interleukin 1 receptor antagonist gene therapy, FX201, is currently developed and progress has been made in a rat model of OA.^240^ It is evident that IL-1β is a potential target for clinical therapy against OA.

TNF-α is up-regulated in OA synovial fluid, synovium, subchondral bone and cartilage. TNF-α has been shown to be involved in a number of pathological advances in osteoarthritis, including inhibition of the production of proteoglycans, connexins, and type II collagen.^241^ Research has reported that high-fat diet could facilitate cartilage damage and up-regulate serum TNF-α level in stress-induced OA mouse model.^242^ Moreover, it was reported that the OA development of mouse model was delayed when TNF-α expression was inhibited.^243^ TNF-α could increase MMP-3, MMP-13, ADAMTS-4, and ADAMTS-5 expression in articular chondrocytes,^244^ and could also induce the production of IL-6.^245^ In a single patient case study of knee OA, subcutaneous administration of adalimumab (monoclonal antibody against TNF-α) obviously reduced synovial effusion and synovitis, and bone marrow edema was virtually eliminated with 6 months of treatment.^246^ However, in a recent randomized trial, adalimumab did not exhibit significantly effective effect on pain symptoms in patients with hand osteoarthritis accompanied by synovitis, compared with placebo.^247^ Therefore, the efficacy and safety of TNF-α antibody therapy against OA need to be confirmed by more basic and clinical experiments.

IL-6 is another critical inflammatory cytokine involved in OA. IL-6 elevation is an independent predictor of knee OA, and high levels of IL-6 can be observed in the joint fluids of OA patients.^248^ A clinical research of OA patients showed that high baseline levels of IL-6 was related to cartilage loss.^249^ In addition, high BMI and increased circulating IL-6 levels were related to the risk of developing knee osteoarthritis.^250^ IL-6 could act as a mediator of HIF-2α to increase MMP-3 and MMP-13 levels.^251^ In OA, IL-6 could also affect synovium, subchondral bone, and muscle of the joint.^252^ In animal experiments, targeted inhibition of IL-6 reduced proteoglycan loss and delayed the progression of OA.^253^ IL-6 inhibitors could also attenuate chondropathy, osteophyte formation and synovitis in OA mouse model.^254^ While the role of IL-6 inhibitors in alleviating OA in animal models is promising, a recent study exploring the role of tocilizumab (IL-6 receptor monoclonal antibody) in alleviating pain of hand OA patients showed that it was no more effective than placebo.^255^ IL-6 might be used as a potential target for basic and clinical trials for osteoarthritis treatment in the future to further explore its effects and mechanism.

**Fig. 3:**
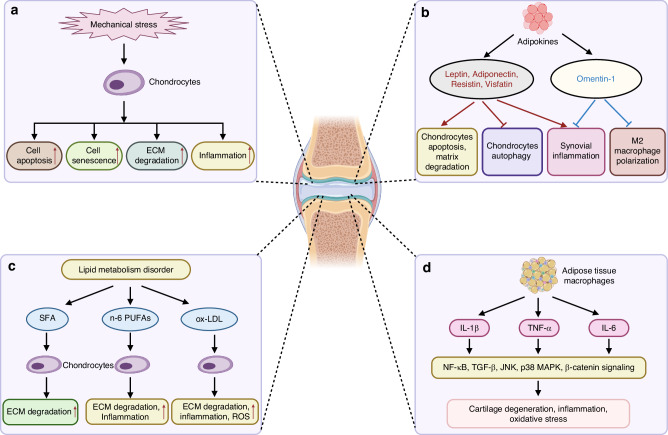
The mechanism of obesity-related factors functioned in osteoarthritis. **a** Mechanical loading promotes OA progression by increasing chondrocytes apoptosis, senescence, ECM degradation and inflammation. **b** Some adipokines including leptin, adiponectin, resistin and visfatin have pro-degenerative effects on the chondrocytes, while omentin-1 have protective effects on the chondrocytes. **c** Lipid metabolism disorder promotes OA progression. Saturated fatty acids (SFAs) could increase chondrocytes ECM degradation. Omega-6 (n-6) PUFA promotes chondrocytes ECM degradation and inflammation. Ox-LDL increases ECM degradation, inflammation, and intracellular reactive oxygen species production. **d** Obesity related inflammatory cytokines, including IL-1β, TNF-α, and IL-6 contribute to OA by targeting NF-κB, TGF-β, JNK, p38 MAPK, and β-catenin signaling pathways

In summary, obesity may contribute to OA progression through mechanical stress, adipokines, lipid metabolism disorder, and inflammatory cytokines (Fig. 3).

## Conclusions and prospects

Accumulating evidence has revealed that while degenerative musculoskeletal diseases are associated with the aging process, obesity has also played essential roles in the pathogenesis and development of degenerative spine and joint diseases. Current studies about the functions and mechanisms of obesity have provided another perspective for understanding the complex pathogenesis of degenerative spine and joint diseases. Obesity could cause an increased level of mechanical loading on the spine disc and weight-bearing joints, contributing significantly to intervertebral disc degeneration and osteoarthritis. Besides, obesity-meditated lipid metabolism disorder, dysregulated pro-inflammatory adipokines and cytokines are deeply involved in the regulation of cell phenotypes and cell fates, ECM metabolism, and inflammation during the pathophysiology of IDD, OSL, and OA.

Correcting lipid metabolism disorders can be a possible strategy for the management of degenerative spine and joint diseases induced by obesity. Lifestyle modifications is a primary and effective treatment for hypertriglyceridemia, and mixed disorders of lipid metabolism which is characterized by raised concentrations of both LDL cholesterol and triglycerides. Administration of statins is the most important pharmacotherapy to lower the LDL-cholesterol concentration, and ezetimibe should be added if the statins treatment alone fails to achieve the target concentration.^256^ Other available pharmacological lipid lowering therapies include PCSK9 inhibitors, fibrates and omega-3 fatty acids. Importantly, statins use has been investigated as therapeutic intervention for osteoarthritis in clinical trials,^257,258^ suggesting the potential feasibility of intervening lipid metabolism disorders to treat degenerative spine and joint diseases.

The roles of adipokines in degenerative spine and joint diseases are complex. Most of the discovered adipokines (e.g. leptin, visfatin, and resistin) are pro-inflammatory to promote the development of these diseases, whereas others are anti-inflammatory to inhibit the disease progression. Although most studies on adipokines are limited to cellular level and in vivo studies in animals currently, research about these adipokines has provided potential therapeutic targets. For instance, applying antibodies to target pro-inflammatory adipokines might be a feasible strategy for treating degenerative spine and joint diseases.

The above obesity-related factors are all critical and plausible links between obesity and degenerative spine and joint diseases. However, research focused on the underlying mechanisms of obesity during the early pathogenesis of degenerative spine and joint diseases is still limited so far, and the specific functions of several specific adipokines have been reported inconsistently. Current knowledge about the major obesity-related factors is still insufficient for clinical practice. Therefore, there remains a need for deeper research to decipher the complex functions and mechanisms of obesity-related factors involved in degenerative spine and joint diseases in the future.
